# A Comparative Study of Thermal Oxidization Resistance of a High-Entropy Metal Boride and a High-Entropy Metal Carbide

**DOI:** 10.3390/ma19132720

**Published:** 2026-06-25

**Authors:** Seth Iwan, Yogesh K. Vohra

**Affiliations:** Department of Physics, University of Alabama at Birmingham, Birmingham, AL 35294, USA; ykvohra@uab.edu

**Keywords:** high-temperature ceramics, thermal oxidation resistance, high-entropy materials

## Abstract

We present a systematic study of thermal oxidation resistance of transition metal borides and carbides up to 1300 °C in a dry air environment. A High-Entropy Metal Boride (HEMB), of composition (Hf_0.2_, Mo_0.2_, Nb_0.2_, Ta_0.2_, Zr_0.2_)B_2_, and a similar High-Entropy Metal Carbide (HEMC) (Hf, Mo, Nb, Ta, Zr)C_5_ were synthesized from precursor mixtures, under 30 MPa of pressure at a temperature of 1800 °C using a Spark Plasma Sintering Device. The synthesized phases were confirmed via X-ray Diffraction analysis, which showed a pure hexagonal AlB_2_-type structure for HEMB and a face-centered cubic (FCC) structure for HEMC, with lattice parameters, a = 3.10 Å and c = 3.37 Å for HEMB and a = 4.524 Å for HEMC. Oxidation resistance was evaluated using a simultaneous thermogravimetric analysis and differential scanning calorimetry (TGA/DSC) stage in which HEMB and HEMC were heated up to 1300 °C at a rate of 2 °C/min in a dry air environment. Scanning electron microscopy (SEM) was used to analyze the resulting oxidized material. Our study demonstrates that HEMB shows better thermal oxidation resistance as compared to a similar metal composition HEMC at high temperatures.

## 1. Introduction

With the ever-increasing demand for materials that can withstand extreme environments, high-entropy metal borides (HEMB) and high-entropy metal carbides (HEMC) have recently emerged as promising materials. Both metal borides and carbides, such as ZrB_2_, HfB_2_, ZrC and HfC_,_ are hard, thermally stable materials [1,2,3,4,5,6,7,8]. However, when multiple metal borides/carbides are combined in equimolar concentration to make a high-entropy material, these desirable properties can be enhanced [9,10,11].

**Figure 1:**
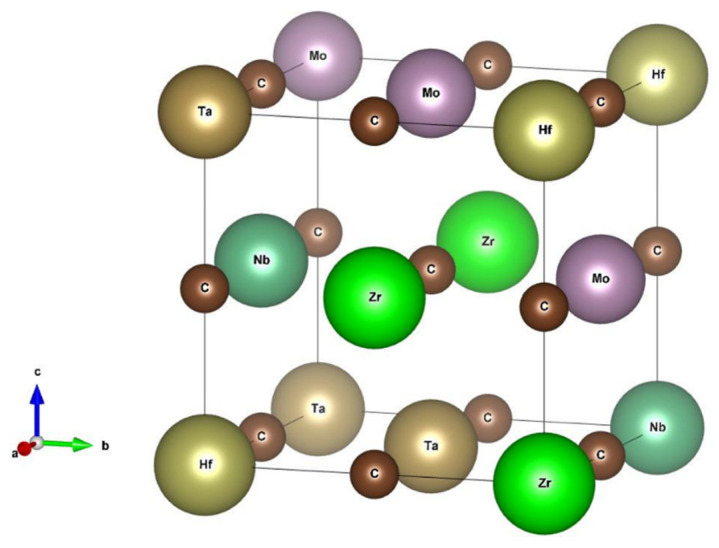
Fm-3m crystal structure of the high-entropy metal carbide.

**Figure 2:**
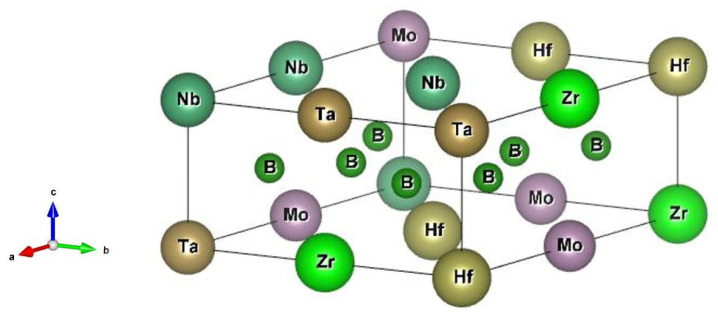
The AlB_2_ type structure represented in a supercell, 2-unit cells in the a and b directions, to highlight the random distribution of metals throughout the crystal structure. The aluminum atoms in the AlB_2_ structure are replaced by Hf, Mo, Nb, and Ta.

This high degree of mixing in high-entropy materials maximizes the configurational entropy, ΔSmix = R ln(N), thereby minimizing Gibbs free energy and resulting in a more stable crystalline phase. HEMCs crystallize in a rock-salt structure (Fm-3m), depicted in Figure 1. HEMBs possess a hexagonal AlB_2_-type structure that retains the boron atom positions while replacing the Al atoms randomly with a chosen composition of metals; in this study, Hf, Mo, Ta, Nb and Zr (Figure 2) were used. Such a high degree of mixing is known to improve physical and mechanical properties of HEMB/HEMC’s and they can achieve better performance than the summation of the individual boride [9,10,11,12,13]/carbide [14,15,16,17,18,19,20,21,22,23,24,25] that make up the high-entropy material.

Thermal oxidation resistance was studied by synthesizing HEMB and HEMC in a Spark plasma sintering device and performing simultaneous thermogravimetric analysis and differential scanning calorimetry (TGA/DSC). The focus of this study was to compare the general oxidation resistance of HEMB and HEMC, composed of similar constituent transition metals.

## 2. Materials and Methods

HEMB and HEMC samples were synthesized using borothermal reduction for HEMB and carbothermal reduction for HEMC. The transition metal oxides are mixed with enough boron/carbon to create HEMB/HEMC and B_2_O_3_/CO. Both byproducts are easily off-gassed at high temperatures during the synthesis process. In this study, the transition metal oxides, HfO_2_, MoO_3_, Ta_2_O_5_, Nb_2_O_5_, and ZrO_2,_ were mixed with boron or carbon powder and then loaded into a tungsten carbide canister with two zirconia milling media. The precursor mixtures were then ball milled together using a SPEX, Metuchen, NJ, USA, 8000M MIXER/MILL, for a total of six hours, pausing every hour to let the machine cool down for 10 min. The material was wet ball-milled, with enough acetone to turn the powder into a thick slurry. After mixing, the sample was passed through a #400 mesh sieve [11,26]. The following chemical reactions occur during the synthesis of HEMB and HEMC samples.

HfO_2_ + MoO_3_ + 1/2 Nb_2_O_5_ + 1/2Ta_2_O_5_ + ZrO_2_ + 18B→ (Hf, Mo, Nb, Ta, Zr)B_10_ + 4B_2_O_3_ (1)

HfO_2_ + MoO_3_ + 1/2 Nb_2_O_5_ + 1/2Ta_2_O_5_ + ZrO_2_ + 17C→ (Hf, Mo, Nb, Ta, Zr)C_5_ + 12CO (2)

**Figure 3:**
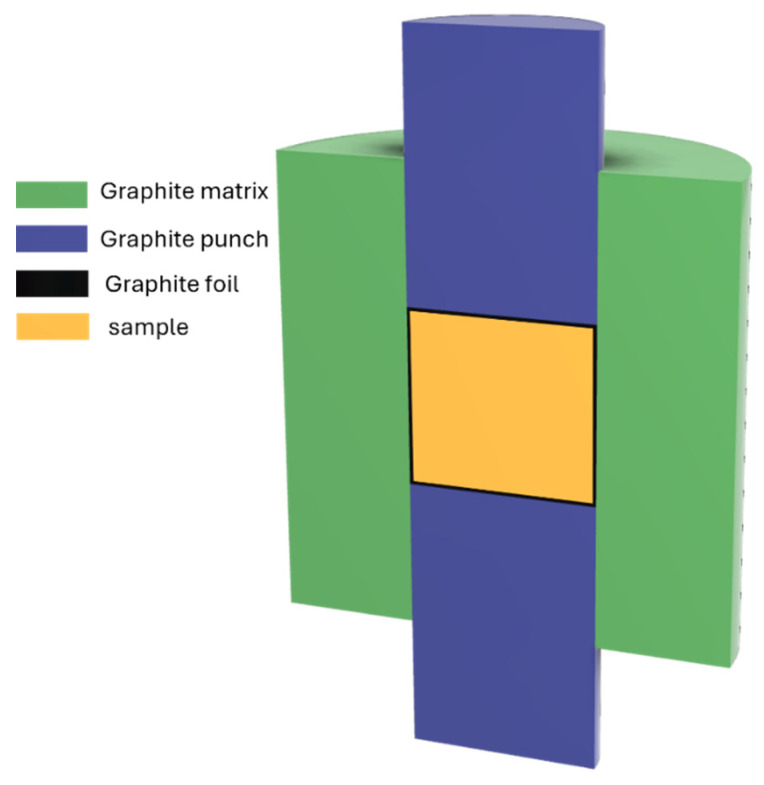
Cross-section of graphite die, and sample assembly used in the Spark Plasma Synthesis system for materials synthesis and densification. The graphite punch used was 25 mm in diameter.

Both synthesis and densification were conducted in a single step using the Spark Plasma Sintering (SPS) device DR. FRITSCH, Fellbach, Germany, FAST/SPS Sintering Press DSP 507. A high-grade graphite die, with a punch diameter of 25 mm, is utilized to compress and heat samples, reaching ~30 MPa and 1800 °C. Figure 3 shows a cross-section of the graphite die assembly used to synthesize the high-entropy ceramics.

The SPS system’s vacuum chamber has two graphite blocks that function as both the anode and cathode, and the pressure plates for the hydraulic press. The graphite die is wrapped in a graphite blanket for better heat retention and placed between the graphite blocks. Fast heating rates are achieved, up to several hundred °C/minute, by passing current through the graphite die, which acts as both mold and a heating element. Temperature is measured via a pyrometer that has optical access from a hole that is drilled halfway through the graphite die matrix.

The graphite die is then compressed to a starting sample pressure of 10 MPa, the vacuum chamber is evacuated and the N_2_ gas is introduced into the system to protect both the sample and the graphite components from oxidation. Once starting conditions are met, the applied pressure is increased to achieve a sample pressure of 30 MPa, and heating is then turned on to ramp up the temperature to the desired limit within 10–15 min. Upon reaching the desired temperature, the sample was held there for 20 min to ensure complete conversion. The sample is then cooled slowly to 1000 °C, at which point the temperature is held constant while the applied pressure is slowly reduced to the starting pressure. This step helps keep the ceramic samples from fracturing due to sudden decompression. Vacuum is held until the sample is cold enough to be removed from the chamber and recovered from the high-pressure, high-temperature sample assembly.

Recovered samples were cleaned using silicon carbide sandpaper to remove impurities from the surface of the sample and to establish a flat surface for XRD analysis. XRD analysis was done using Panalytical B.V., Almelo, The Netherlands, and the data were analyzed using High-Score plus (version 4.8). This XRD data was used to verify synthesis and the purity of the samples by confirming FCC and hexagonal structures for HEMC and HEMB, respectively, and the absence of any secondary phases.

Oxidation measurements were performed with a Linseis PT1600 TGA/DSC stage, Linseis Messgeräte GmbH, Selb, Germany. This system can reach 1600 °C in a controlled gas environment while collecting simultaneous thermogravimetric analysis (TGA) and differential scanning calorimetry (DSC) data. Crucibles are held aloft in the furnace by a long, thin alumina rod which is connected to a high-precision balance. TGA data is collected from the balance and the DSC sensor, measuring the microvolt difference between the sample and reference crucible thermocouples. This DSC sensor is calibrated by using the melt data of indium and gold. The desired heating profile is run four to five times with both crucibles empty, resulting in a zero curve. This zero curve accounts for the long alumina stem wobbling during heating, by subtracting the false mass change caused by the change in the moment of the balance fulcrum.

Prepared samples are loaded into the TGA stage, and a dry air environment is established. This is accomplished by evacuating the furnace chamber and purging with dry compressed air two to three times. The vacuum pump is turned off, and dry compressed air is continually pumped through the furnace chamber to continually replenish the air supply. The sample can then be put through the desired heating profile.

## 3. Results and Discussion

**Figure 4:**
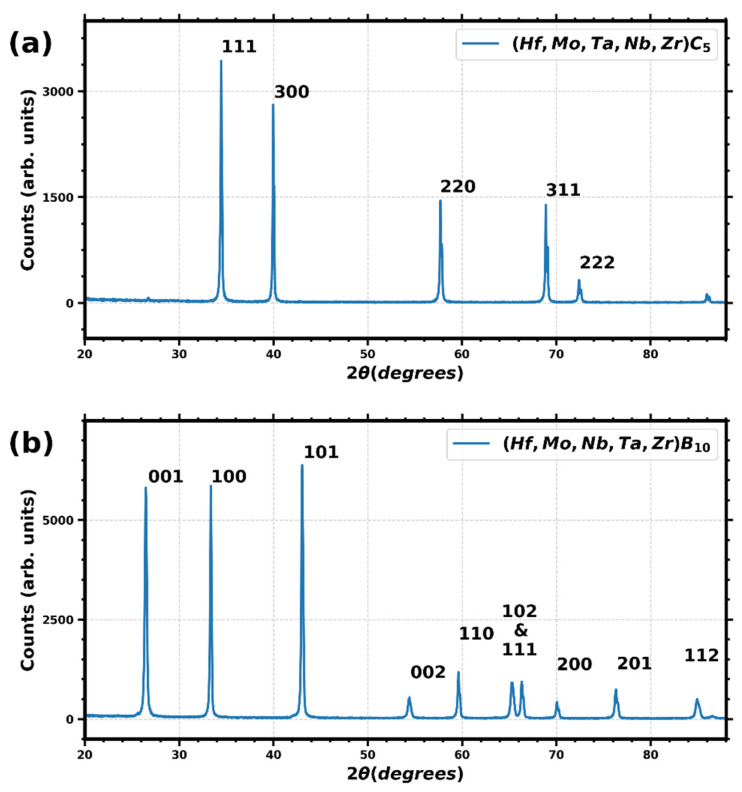
XRD analysis of the HEMC (**a**) and HEMB (**b**) samples after synthesis under 30 MPa and 1800 °C. Both samples show a single-phase material without any contamination of secondary phases.

The HEMC and HEMB synthesized samples are demonstrated to be pure from their single-phase XRD patterns. Fits of the XRD give lattice parameters of a = 3.10 Å and c = 3.37 Å for the P6/mmm HEMB and a = 4.524 Å for the Fm-3m HEMC, Figure 4. Samples were sectioned into cubes and polished to 600 grit before TGA/DSC analysis. Three HEMB samples of similar size were used along with one larger piece to get a better grasp of the sample sizes’ effect on the TGA data. Two HEMC samples were used for TGA/DSC and a larger sample was put through a similar heating cycle to verify results.

**Figure 5:**
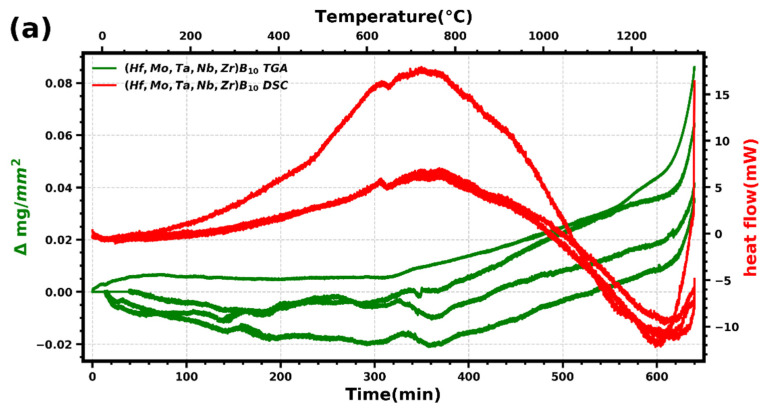

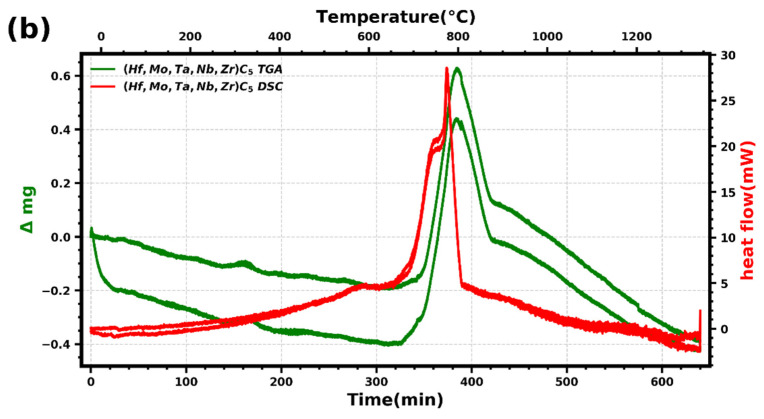
TGA/DSC analysis of the HEMB and HEMC samples. samples were heated at 2 °C per minute up to 1300 °C. The HEMB (**a**) sample is normalized by surface area, whereas HEMC (**b**) is not, due to the sample fully crumbling to powder.

**Figure 6:**
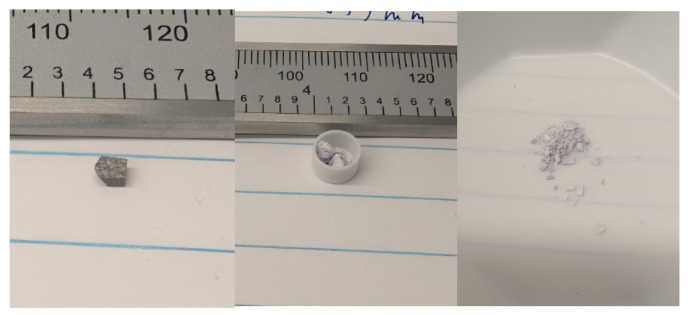
A large piece of HEMC that underwent the same heating conditions as the TGA/DSC samples, to show that the complete oxidation was not due to the smaller sample size. Leftmost is HEMC before TGA/DSC, middle is the HEMC sample right after TGA/DSC and the rightmost is the HEMC sample after TGA/DSC, which just crumbled after being slightly prodded.

The TGA/DSC heating profile of 2 °C/minute is used to get an overview of how well these materials react over a wide range of temperatures. It is immediately apparent that HEMC reacted very differently from HEMB, Figure 5. The HEMC samples were completely oxidized and were turned to a powder by the end of the run, Figure 6; this is why the HEMC data is not normalized by surface area. The sudden spike in the TGA signal at 700 °C for the HEMC indicates that the sample oxidizes rapidly, while the HEMB has a slow increase in the TGA data. Both sets of TGA data are backed up by the corresponding DSC signal, a lack of a peak indicating a slow oxidation and a sharp peak for a sudden oxidation. One might notice that the DSC signal seems to lead the TGA signal, but the time derivative of the TGA data produces peaks that coincide with DSC signals.

**Figure 7:**
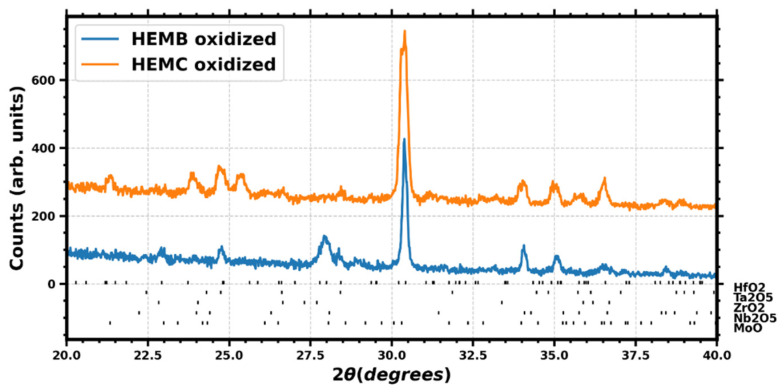
XRD analysis of HEMB and HEMC after TGA/DSC analysis. Common forms of each transition metal oxide are shown to demonstrate the complexity of the spectra.

**Figure 8:**
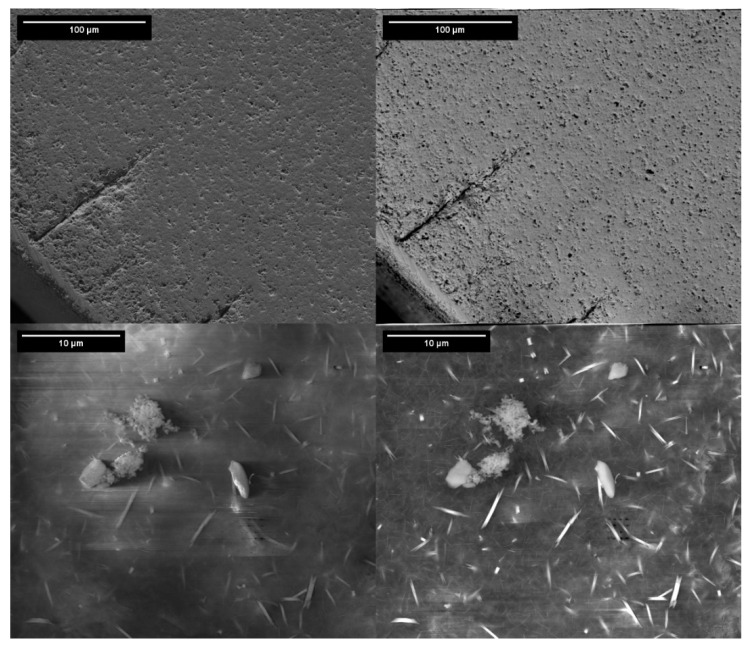
SEM image of HEMB after 1300 °C. The right column is the backscattered images. The top row is a section HEMB sample, while the bottom row shows a magnified image of the oxide film. The cross-section HEMB sample shows the oxide layer in the bottom left corner, moving toward the top-right corner shows crevasses, which is the more porous HEMB region, and then finally the untouched bulk of the HEMB sample, which starts where crevasses end.

XRD analysis of both samples after TGA/DC, Figure 7, reveals some similarly looking peaks but also some peaks that are unique to each sample. Similar peaks, like the strong peak just above 30°, indicate the formation of similar oxides, while the different peaks are presumed to be some compound using either boron or carbon. Having the same constituent transition metals in both HEMB and HEMC, similar transition metal oxides are expected, and their common forms are indexed below the XRD spectra. XRD analysis on HEMC and HEMB shows no signs of their previous crystal structure. For HEMC, which had completely decomposed and crumbled, it was easy to spread thinly on the silicon wafer for XRD, showing that none of the HEMC sample remained. A much larger HEMC piece underwent similar heating conditions. Figure 6 visually backs up the TGA/DSC results of complete oxidation. HEMB, on the other hand, retained its bulk form; however, the oxide layer is too thick for sufficient X-rays to penetrate and diffract off the HEMB sample, resulting in no HEMB hkl peaks in Figure 7. Subsequent sectioning of HEMB cuts the sample in half, allowing X-rays to diffract off the HEMB material and reveal the hexagonal AlB_2_-type structure that was hidden by the oxide layer. This oxide layer can be seen in the SEM images of both secondary electrons and back scattered electron, in the bottom left corner of the top row of Figure 8. The back-scattered image helps differentiate the outer boron oxide film from the dense oxide film layer.

Four main regions are identified using EDS. The first region is mostly a boron oxide layer, followed by the dense oxide layer, which is the dense region in the bottom left corner of the HEMB cross-section sample in Figure 8. The third region is made up of a HEMB that is more porous than the bulk and has developed crevasses that act as a route for oxygen to penetrate the sample and for oxides to move outwards. The fourth region is the untouched bulk HEMB material. SEM on HEMB’s oxidized surface after 1300 °C shows the formation of spear-like formations, which are revealed to be a high Z material when using backscattered SEM. EDS spot analysis of a few spears gives boron, oxygen and molybdenum, indicating that the spears are molybdenum or molybdenum oxide crystals growing through the boron oxide layer. No SEM was done on the HEMC samples due to the samples completely oxidizing and crumbling into a powder.

The stark difference between the HEMB and HEMC was surprising. HEMC had a sudden onset of oxidation indicated by the sharp increase in DSC and TGA signals, followed by a continuous loss of mass for the rest of the run. This mass loss has an inflection point at 875 °C, where the slope of mass loss is decreased. By the end of the run, HEMC had completely crumbled into a powder. HEMB, on the other hand, demonstrated a very slow rate of oxidation, indicated by the small slope in TGA and a lack of a peak in DSC signals. Both HEMC and HEMB are stable until 650 °C. The presence of a boron oxide layer helps protect the HEMB sample, but there seems to be no type of protective layer to protect the HEMC sample. HEMC was expected to do better as a similar composition with Ti instead of Mo showed decent promise [22,23]. Another study on HEMCs looked at a combination of TaTiNbZr with the fifth transition metal being Mo, Hf or W, in which only the W-containing sample had any mass loss seen in TG curves [19].

HEMB’s better oxidation resistance is helped by the boron oxide film, which acts as another layer that oxygen has to penetrate. In the SEM images of HEMB’s oxidized surface, Figure 8 row 2, the small spears are rich in molybdenum according to EDS. This could indicate that the boron oxide film was slowing down the volatilization of molybdenum oxide that may have been occurring in HEMC. This thought is supported by the HEMC TGA data decrease beginning at ~800 °C, which is around the reported melting point of MoO_3_. However, the cause of HEMC decomposition seems to be a lot more complicated, as the other aforementioned HEMCs, with slightly different compositions, did not have this complete failure. The variability in thermal oxidation behavior of various HEMCs in the literature would require future investigations to clarify the role of chemical composition and materials processing conditions in controlling oxidation at high temperatures.

## 4. Conclusions

The high-phase-purity samples of HEMB and HEMC were synthesized by spark plasma sintering for a direct comparison of their thermal oxidation resistance. Both HEMB (Hf_0.2_, Mo_0.2_, Nb_0.2_, Ta_0.2_, Zr_0.2_)B_2_ and HEMC (Hf, Mo, Nb, Ta, Zr)C_5_ were thought to be materials with better thermal properties than normal borides and carbides; as such, it was of interest to study how HEMB and HEMC of the same constituent metals compared. The results of TGA/DSC studies in dry air to 1300 °C revealed HEMC’s poor oxidation resistance. HEMC oxidized rapidly at ~670 °C and by the end of the study had completely crumbled. HEMB, on the other hand, showed excellent oxidation resistance, with oxidation onset happening at ~660 °C and reaching an average normalized mass gain of only 0.057 mg/mm^2^. Out of HEMB (Hf_0.2_, Mo_0.2_, Nb_0.2_, Ta_0.2_, Zr_0.2_)B_2_, and HEMC (Hf, Mo, Nb, Ta, Zr)C_5,_ investigated in this study, only HEMB showed thermal oxidation resistance acceptable to be employed in extreme temperature environments up to 1300 °C.

## Data Availability

The original data presented in the study are openly available on FigShare at https://doi.org/10.6084/m9.figshare.32348103.
